# Is there any association between the presence of biomarkers and apical periodontitis? A systematic review

**DOI:** 10.3389/fimmu.2024.1366954

**Published:** 2024-05-22

**Authors:** José Mário Matos-Sousa, Victória Santos Chemelo, Deborah Ribeiro Frazão, Leonardo Oliveira Bittencourt, João Daniel Mendonça de Moura, Caio Melo Mesquita, Guido Marañón-Vásquez, Nathalia Carolina Fernandes Fagundes, Luiz Renato Paranhos, Lucianne Cople Maia, Marta Chagas Monteiro, Rafael Rodrigues Lima

**Affiliations:** ^1^ Laboratory of Functional and Structural Biology, Institute of Biological Sciences, Federal University of Pará, Belém-Pará, Brazil; ^2^ Department of Preventive and Social Dentistry, Faculty of Dentistry, Federal University of Uberlândia, Uberlândia, Minas Gerais, Brazil; ^3^ Department of Pediatric Dentistry and Orthodontics, School of Dentistry, Federal University of Rio de Janeiro, Rio de Janeiro, Brazil; ^4^ School of Dentistry, Faculty of Medicine and Dentistry, University of Alberta, Edmonton, AB, Canada; ^5^ Health Science Institute, Federal University of Pará, Belém-Pará, Brazil

**Keywords:** apical periodontitis, biomarkers, inflammatory markers, systemic biomarkers, and endodontic infection

## Abstract

This systematic review aimed to verify whether there is evidence of an association between apical periodontitis and the presence of systemic biomarkers. This study adhered to the Preferred Reporting Items for Systematic Reviews and Meta-Analyses - PRISMA. For this, the acronym PECO was used; population (P) of adult humans exposed (E) to the presence of apical periodontitis, compared (C) to adult humans without apical periodontitis, and the outcome (O) of the presence of biomarkers was observed. The articles were searched in PubMed, Scopus, Web of Science, LILACS, Cochrane Library, OpenGray, and Google Scholar grey databases. Subsequently, studies were excluded based on title, abstract, and full article reading, following the eligibility criteria. The methodological quality of the selected studies was evaluated using the Newcastle-Ottawa qualifier. After exclusion, 656 studies were identified, resulting in 17 final articles that were divided into case-control, cross-sectional, and cohort studies. Eight studies were considered to have a low risk of bias, one had a medium risk of bias, and eight had a high risk of bias. In addition, 12 articles evaluated biomarkers in blood plasma, four evaluated them in saliva, and only one evaluated them in gingival crevicular fluid. The results of these studies indicated an association between apical periodontitis and the systemic presence of biomarkers. These markers are mainly related to inflammation, such as interleukins IL-1, IL-2, and IL-6, oxidative markers, such as nitric oxide and superoxide anions, and immunoglobulins IgG and IgM.

**Systematic review registration:**

https://www.crd.york.ac.uk/prospero/, identifier (CRD42023493959).

## 1 Introduction

Because of microbial infection of the root canals, apical periodontitis (AP) is a chronic inflammatory disease that can destroy periradicular tissues (1). AP has microbial factors as its main etiology and is involved in the initiation, development, and persistence of the disease, sustained by a biofilm that can invade periodontal structures (2, 3).

AP development is based on the inflammatory response and bone destruction in periapical tissues related to the microbial location within the root canal, organization of the biofilm, and degree of virulence (3, 4). Microorganisms can cause direct tissue damage and modulate host immune responses by secreting products, including enzymes, immunoglobulins, cytokines, chemokines, the RANK/RANKL/OPG system, and other inflammatory markers (5, 6).

During AP development, acute and chronic inflammatory reactions can develop depending on the intensity of the bacterial infection and the host immune response (6). The large amount and interaction of various inflammatory stimuli can influence and alter disease state and progression (5). The host immune response attempts to localize the infection and prevent its further spread at the expense of disrupting the apical periodontal tissue involving the periodontal ligament, root cementum, and alveolar bone (7).

The periradicular region is modulated by proinflammatory and anti-inflammatory biomarkers. Most of these biomarkers are regulated in response to bacterial infection. The balance between pro- and anti-inflammatory biomarkers controls the host immune response to antigen stimulation during chronic inflammation, triggering the defense process and preventing bone resorption (8, 9).

In apical periodontitis, biomarkers are found in various samples, such as gingival crevicular fluid, plasma, periapical exudate, serum, and saliva. Pro-inflammatory biomarkers are produced mainly by TH1 cells, macrophages, and neutrophils, such as interleukin (IL) -1β, IL-6, interferon (IFN)-γ, and tumor necrosis factor (TNF)-α. In contrast, anti-inflammatory biomarkers are released by TH2 and Treg cells, such as transforming growth factor (TGF)-β and IL-4 (8, 9).

Considering the influence of biomarkers on inflammation and their roles in apical periodontitis, it is important to evaluate the association between apical periodontitis and the presence of biomarkers. Therefore, this systematic review aimed to gather scientific evidence to analyze the association between the presence of systemic biomarkers and apical periodontitis in adult humans.

## 2 Material and methods

### 2.1 Register and protocol

This systematic review was registered on the platform responsible for the records and organization of systematic reviews, PROSPERO, under code CRD42023493959. Additionally, this systematic review was conducted according to the Preferred Report Items for Systematic Reviews and Meta-Analyses (PRISMA) 2020 version and according to the Conducting Systematic Reviews and Meta-Analyses of Observational Studies of Etiology (COSMOS-E) guideline (10).

### 2.2 Eligibility criteria and search strategy

This systematic review aimed to determine whether patients with apical periodontitis have different concentrations of biomarkers than healthy patients. We utilized the PECO acrostic to define the eligibility criteria, in which “P” represents the population, “E” the exposure, “C” the comparison, and “O” the outcome. This review used P: human, E: apical periodontitis, C: absence of apical periodontitis, and O: presence of biomarkers. The study included only observational studies and excluded case reports, reviews, opinion articles, animal studies, and *in vitro* studies. Two examiners (JMMS and VSC) selected the studies by consulting a third examiner (RRL) in cases of disagreement.

The search strategy was performed using MeSH and Entry terms for searching in the following online databases: MEDLINE (PubMed), Scopus (https://www.scopus.com/), Web of Science (https://www.webofscience.com/), Lilacs (BVS), and EMBASE (https://www.embase.com/), using the search string: “Apical Periodontitis” AND “Biomarkers,” as the main meSHs, however, the search key will be presented in **Supplementary Table 1**. We also searched Google Scholar and OpenGrey (June 2022) using the anonymous guide as the grey literature using the only search string: “Apical Periodontitis” AND “Biomarkers.” There were no restrictions on the language or year of publication. Searches were conducted between January 2022 and January 2023. The studies found in each database were exported in the order of the search to a reference organization application (EndNote^®^, version X9, Thomson Reuters, Philadelphia, USA).

### 2.3 Study selection process and data extraction

Two examiners (JMMS and VSC) independently performed the data extraction. A third reviewer was consulted in case of disagreement. This process began with the automatic and manual deletion of duplicates after the articles were imported. Subsequently, studies were evaluated based on their titles and abstracts. These findings were assessed thoroughly. Furthermore, the references of the included studies were manually checked to select all published articles that met the inclusion criteria.

After selecting the final articles, we extracted relevant data for a systematic review. The relevant extracted data were related to the author’s name, year of publication, country, type of study, analysis material, participants (number and mean age of the sample), evaluation of biomarkers (evaluated biomarker and evaluation method), evaluation of apical periodontitis (form of diagnosis, symptomatology, and extent of the lesion), statistical analysis, and results.

### 2.4 Risk of bias

#### 2.4.1 Quality assessment and risk of bias

Two reviewers (DRF and LOB) used the Newcastle-Ottawa Scale methodology, developed in collaboration between the Universities of Newcastle, Australia, and Ottawa, Canada, to assess the methodological quality and risk of bias of the included studies. This tool was developed to evaluate the quality of nonrandomized research by incorporating quality judgments into the interpretation of meta-analytic findings.

A “star system” was created as part of this protocol. The studies were evaluated by two reviewers from three perspectives: selection of study groups, group comparability, and verification of exposure or outcome of interest for case-control or cohort studies. This checklist aimed to establish an instrument that provides an easy and convenient tool for assessing the quality of nonrandomized studies in a systematic review (12) (**Supplementary Table 2**).

For cross-sectional investigations, we used Peinado et al.'s (11) adaption of the Newcastle-Ottawa Scale procedure, which still uses the “star system.” After the qualitative assessment, the number of “stars” is used to calculate each study’s risk of bias (**Supplementary Table 2**).

#### 2.4.2 Evaluation of control statements for possible confounders and bias consideration

This evaluation was based on the results of a previous study by Hemkens et al. (13). All eligible studies were first analyzed for explicit mention of adjustment analyses to control for possible confounders. Studies were excluded if they did not report or if the analyses were unclear. Furthermore, the remaining studies were critically appraised by two independent reviewers (CMM and LRP), and disagreements were resolved by a third reviewer (RRL). Six previously established questions (**Table 1**) were used to evaluate the abstracts and discussions of each remaining study. The sixth question evaluated the conclusion section; in cases lacking a specific conclusion, the last paragraph of the Discussion section was considered.

**Table 1:** Evaluation of control statements for possible confounders and bias consideration.

Section	Question	Possible answers with explanation	N (%)
Abstract and Discussion	Is the term “confounding” mentioned in Abstract or Discussion?	**Specific:** if authors used the exact term “confounding.”	2 (40%)
		**Alluded:** if authors used a similar term or phrase.	1 (20%)
		**No:** if the authors used neither the exact nor similar term.	2 (40%)
	Is the term “bias” used in Abstract or Discussion?	**Yes:** if authors used the term “bias.”	1 (20%)
		**No:** if authors did not use this term.	4 (80%)
	Is any specific mention about non-adjusted variables in Abstract or Discussion?	**Yes:** if there was specific mention about non-adjusted variables with no reasons presented.	2 (40%)
		**Not measured:** if there was specific mention about non-adjusted variable not being measured.	2 (40%)
		**Other reasons:** if there was specific mention about non-adjust variables and with plausible reasons for not adjusting them.	0
		**No reasons:** if there was specific mention about non-adjusted variables and with implausible reasons for not adjusting them.	0
		**No:** if there was no mention about any non-adjusted variable.	1 (20%)
	Is there any mention about confounders affecting results in Abstract or Discussion?	**Likely:** if authors used terms such as “likely” or convincing statements that confounders were not controlled.	0
		**Possibly:** if authors used terms such as “possibly” or unsure statements that confounders were or were not controlled.	5 (100%)
		**Unlikely:** if authors used terms such as “unlikely” or convincing statements that confounders were controlled.	0
		**No mention:** if there was no mention about this possibility.	0
	Is there any statement about the need for caution in interpreting the results?	**Yes:** if there was explicit mention about the need for caution in interpreting the results obtained in the study.	5 (100%)
		**No mention:** if there was no mention about this need for caution.	0
Conclusion	Does Conclusion include any limitation about confounders?	**Yes:** if there was a mention about this limitation.	1 (20%)
		**No:** if there was no mention about this limitation.	4 (80%)

#### 2.4.3 Assessment of confounding factors

This assessment was based on previous studies by Wallach et al. (14). The remaining studies in the final evaluation was assessed using the Methods and Results section. Assessment of confounding factors was performed by two independent reviewers (CMM and RLP) and a third reviewer (RRL) to resolve conflicts. They identified the variables and confounding domains for each study. The variables were classified into three groups: (1) adjustment (used in multivariate analysis or Poisson regression to control for possible confounders), (2) stratification (used in sample selection to create strata), and (3) matching variables (used to pair known characteristics between study participants or groups).

### 2.5 Quantitative analysis (meta-analysis)

Quantitative analysis could not be performed since there was a significant divergence in the methodologies of each study, in addition to the diversity of biomarkers evaluated in each study and the different analytical materials.

### 2.6 Level of evidence (GRADE)

A narrative synthesis of the collected data from the included studies was conducted, including body fluids whose biomarkers were evaluated, target population characteristics, and the type of outcome. The Grading of Recommendations, Assessment, Development, and Evaluation (GRADE) was used to compute the absolute expected effect and summary of evidence. The GRADE is a grading system for the evidence level and strength of health recommendations. The mean difference was used as an effect estimate for the Evidence Profile. When serious or extremely serious concerns regarding bias, inconsistency, indirectness, imprecision, and publication bias are identified, the certainty of the evidence drops by one or two. The level of evidence tends to improve when the effect of all plausible confounding factors is minimized or when it suggests a spurious effect.

## 3 Results

### 3.1 Characteristics of the included studies

**Figure 1:**
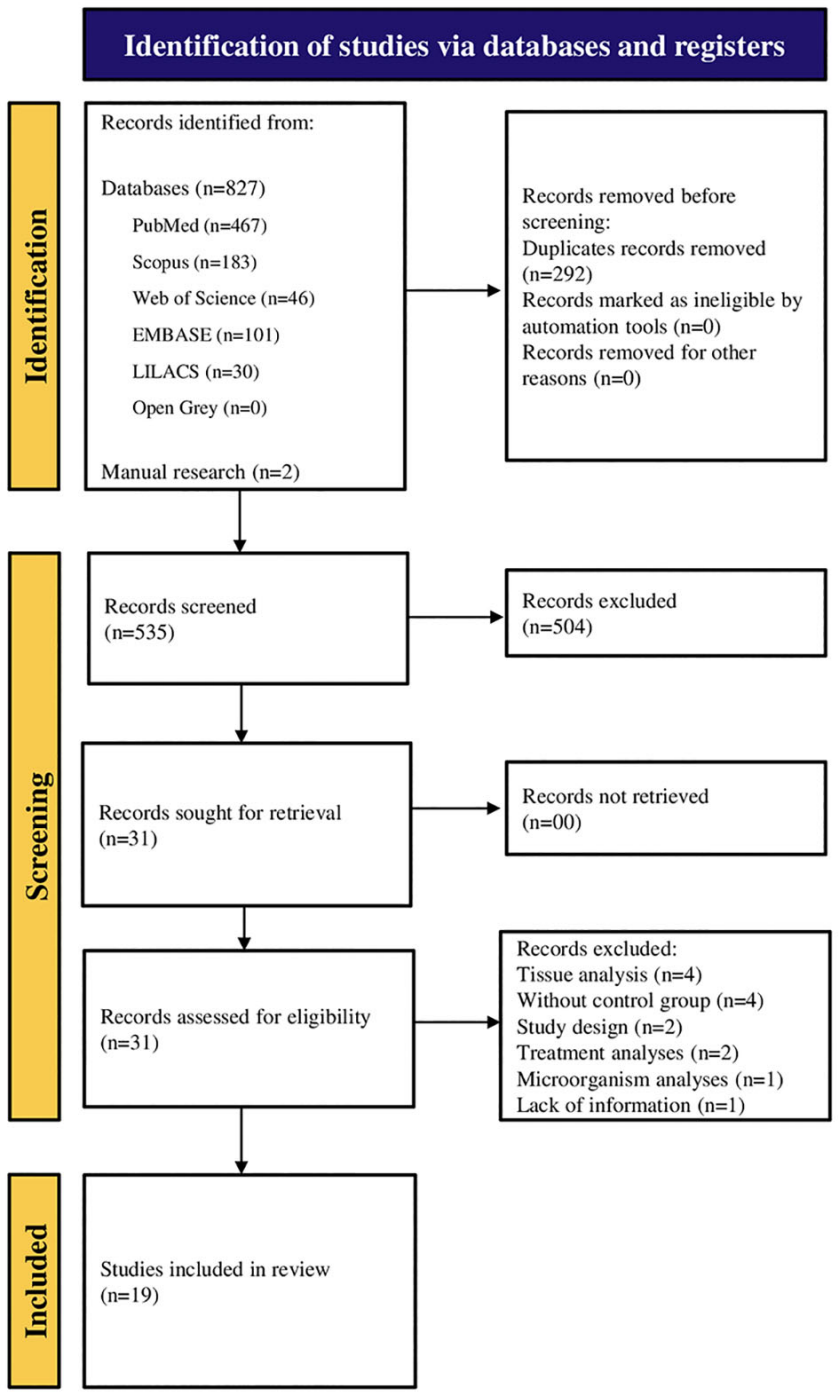
PRISMA flowchart, exclusion process for selecting final articles.

Following the database search, we found a total of 827 studies. After removing duplicates, 535 remained. Following exclusion based on the title and abstract, 31 papers were fully examined. Later, 14 articles were removed because of non-compliance with PECO. For instance, tissue analysis (15–18), absence of a control group (19–22), and non-observational studies (23, 24), treatment analyses (25, 26), microorganism analyses (27) and lack of information (28). Therefore, 17 articles remained in the systematic review (6, 29–44) distributed across cross-sectional, cohort, and case-control studies, as shown in **Figure 1**.

### 3.2 Results of individual studies

**Figure 2:**
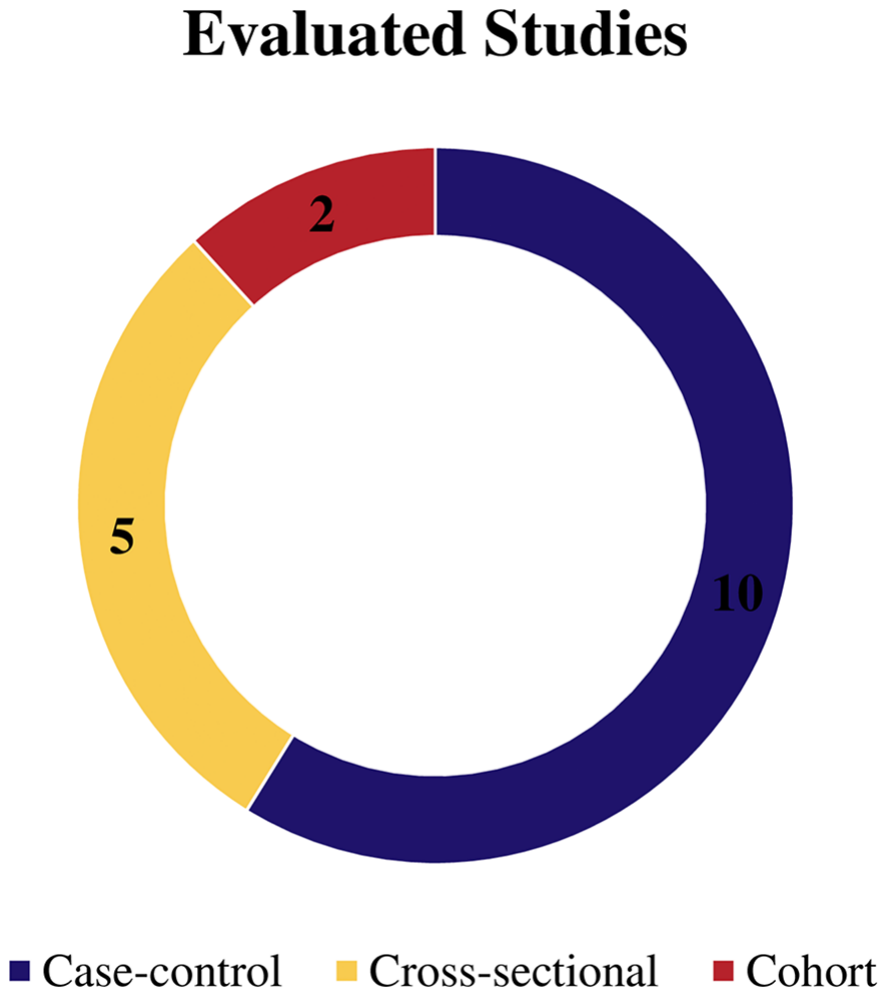
Graph with results of the evaluated study designs.

Among the final 17 studies, the majority were case–control studies which included ten articles (6, 32, 34, 35, 38, 40–44). Five cross-sectional studies (30, 31, 33, 36, 39). Finally, two cohort searches (6, 37), as shown in **Figure 2**.

**Figure 3:**
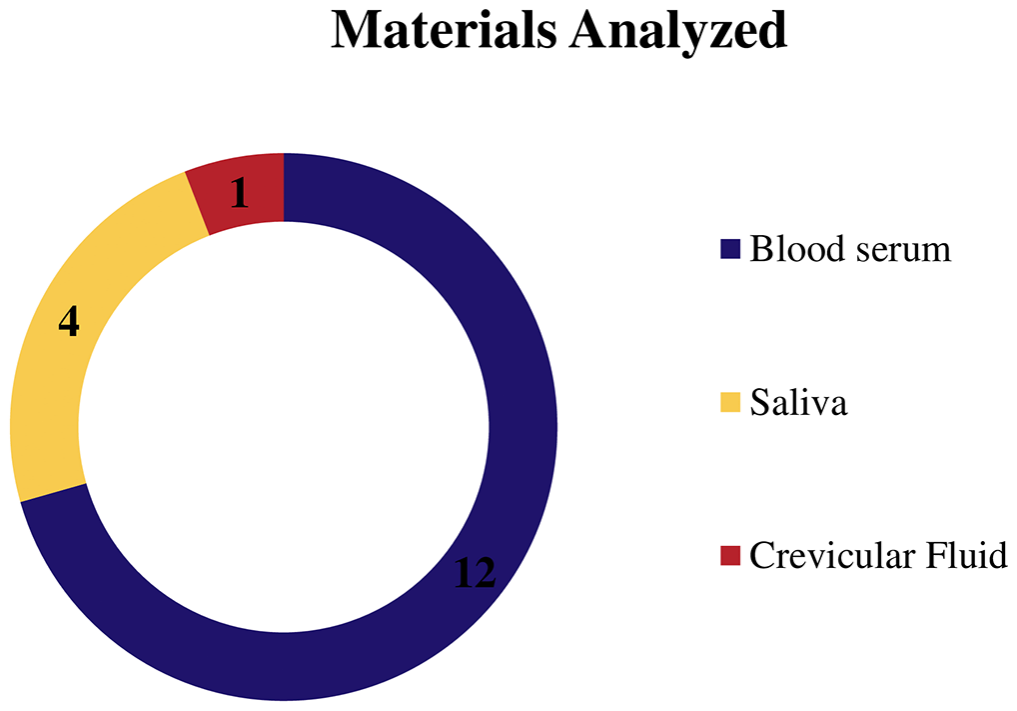
Graph showing the number of articles that evaluated each analysis material.

The materials analyzed in these studies were blood serum, saliva, and crevicular fluid. **Figure 3** shows the ratio of the number of articles for each material. The main material analyzed was blood plasma, with 12 articles (29, 31–35, 37, 38, 40, 41, 41, 2020; 43). Saliva was evaluated in four studies (6, 36, 42, 44), and only one study (30) evaluated the gingival crevicular fluid.

The average sample size was ± 94.56, with a combination of males and women. The form of biomarker evaluation was primarily verified by the ELISA test in nine of the selected studies (6, 29–31, 33, 38, 39, 41, 44), with the remaining eight alternating between spectrophotometrically, system cadmium-copper reagent, glomax luminomete (35); enzyme immunoassay (EIA) kits (36 and 32); Biological antioxidant potential (BAP) test and d-ROMs test (37); Gauging system (40); Agilent 5977B interfaced to the GC 7890B (42), radioimmunodiffusion (43) and immunoassay customized kits (34).

**Figure 4:**
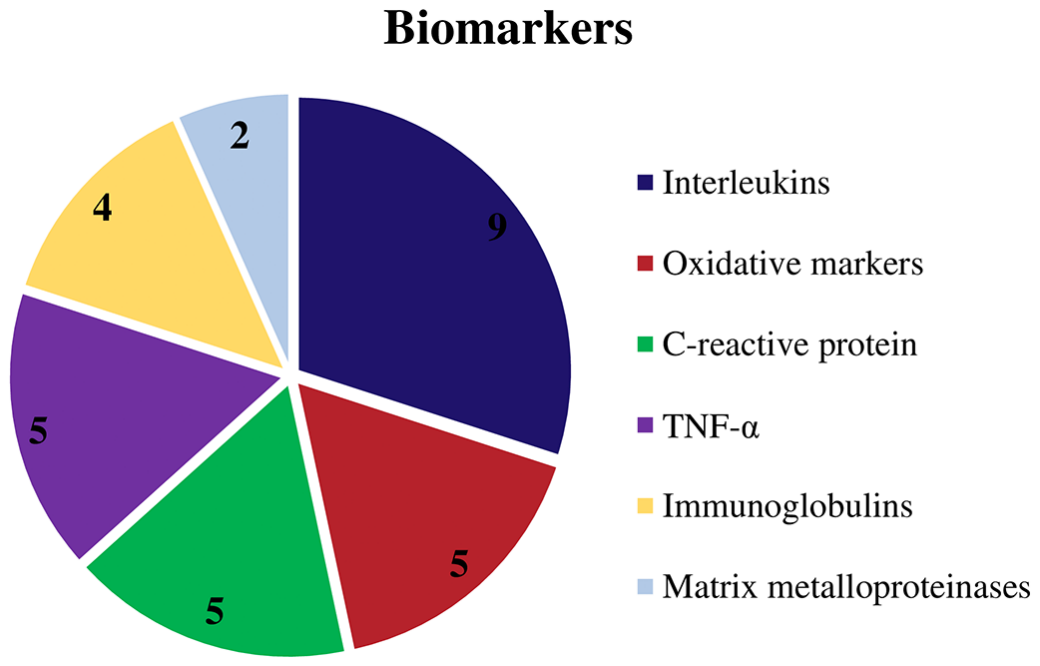
Graphic showing the biomarkers evaluated by the authors.

Among the biomarkers examined, interleukins were found in 9 research (29, 30, 32, 31, 33, 34, 36, 39, 41), followed by oxidative markers in 5 studies (30, 35, 37, 40, 44), also C-reactive protein (CRP) in 5 articles each (33, 34, 36, 38, 41) with the same number of articles are analyzed TNF-a (30–32, 34, 41), and immunoglobulins in 4 articles (6, 33, 38, 43) in addition, matrix metalloproteinase, lipoproteins, lipids, were found, as shown on **Figure 4**. **Table 2** displays all the individual qualities.

**Table 2:** Summary of the characteristics and results of the included studies.

Sample: Blood/Serum							
Author, Country, Year and Study Design	Participants		Biomarker Evaluation		Apical Periodontitis		Results
	Sample size	Age mean (SD) in years	Biomarker	Evaluation Method	Diagnostic Method	Clinical Diagnosis	
Georgiou et.,/Netherlands**/**2023**/**Case-control	Case: 27 Control: 26	Case: SD 14.5 Control: SD 13.17	VEGF, G-CSF, IL-1α, IL-17A IL-1β, IL-4, IL-6, IL-8, IL-10, IL-12p70, IFN-γ, TNF-α CRP, MIP-1α OPG RANKL	Immuno kit: customized V–plex cytokine panel 1 Immuno kit: customized V–plex Proinflammatory panel 1 Immuno kit: custom-made V–plex Vascular Injury panel 2 and a Chemokine panel 1 Human OPG antibody set in combination with MSD GOLD Small Spot Streptavidin Magnetic bead-based singleplex assay (# LXSAHM; R&D Systems)	Clinical and radiographic examination	Asymptomatic apical periodontitis	Significant differences were found for GM-CSF, IL-1β, and IL-4, with concentrations in the apical periodontitis subjects being lower than in the control subjects.
Sirin et al.,**/**Turkey**/**2021**/**Cross-sectional	Case: 121 Control: 45	Case: 39 ± 18 years Control:41 ± 15 years	IL-6 PAPP-A	Human IL-6 ELISA kit PAPP-A ultrasensitive (us) ELISA kit	Clinical and radiographic examination	Asymptomatic apical periodontitis	IL-6 revealed that, although there was no difference between the healthy group and grade 1 (smallest AP lesion size) (p>0.05), the differences between other grades were statistically significant (p<0.05).
Bergandi et al,./2019**/**Italy**/**case-control	Case: 23 Control: 20	Case: 33.05 ± 6.27 Control: 32.07 ± 5.28	IL-1, IL-6, TNF-α, ET-1, ICAM-1/CD54, sVCAM-1/CD106, sCD14 and E-selectin	EIA kit	Clinical and radiographic examination	NR	The groups did not differ significantly in clinical parameters or markers of systemic inflammation, except for higher concentrations of IL-1 and sCD14 in the apical periodontitis group compared to the control group. Additionally, concentrations of ET-1, ICAM-1/CD54, and E-selectin were significantly higher in apical periodontitis patients compared to controls.
Garrido et al./2019**/**Chile**/**Cross-sectional	Case: 27 Control: 28	18-40 years	HsCRP IgG IL-6, IL-10, IL-12p70, MMP-8, sVCAM-1, sICAM-1, and sE-selectin	Blood test IgG levels were quantified by a commercial ELISA kit. It is quantified by multiplex panels in a Luminex platform and analyzed with MILLIPLEX Analyst software	Clinical and radiographic examination	Asymptomatic apical periodontitis	HsCRP levels were significantly higher in patients with apical periodontitis versus controls (median = 2.54 vs 0.78). Also, the levels of IL-6, matrix metalloproteinase 8, and soluble E selectin were significantly higher in patients with apical periodontitis.
Sirin et al.,**/**Turkey**/**2019**/**Case-Control	Case: 104 Control: 40	18-60 years	HsCRP	Levels of the serum are measured with the ELISA test.	Clinical and radiographic examination	Symptomatic apical periodontitis	hsCRP levels in patients with an apical periodontitis Grade 2 and 3 were higher than both apical periodontitis Grade 0 and 1 (p<.05).
Gomes et al.,**/**Brazil**/**2017**/**Case Control	Case: 24 Control: 23	≥18 years	Oxidation protein products (AOOP); PON1 total activity; Total radical trapping antioxidant parameter (TRAP). Nitric oxide (NO) metabolites (NOx), Hydroperoxides (LOOH); Sulfhydryl (−SH) group;	The quantification measure of AOPP and the plasmatic activity of PON1, NOx, and TRAP, were done by a spectrophotometrically used microplate reader. NO levels were assessed indirectly by determining the plasma nitrite concentration mediated by the system cadmium-copper reagent. LOOH was determined by photon emission during the formation of lipid hydroperoxides in a Glomax luminometer. SH groups from proteins were evaluated in a spectrophotometer Helios α.	Clinical and radiographic examination	Asymptomatic apical periodontitis	Root canal LPS was significantly higher in those with chronic apical periodontitis (141.2 ± 14.4 EU/mL) than in those without (90.5 ± 16.4 EU/mL).
Kimak et al.,**/**Poland**/**2015**/**Case-Control	Case: 43 Control: 20	Case 1: ≥50 years Case 2: <50 years Control: 23-50 years	Lipids Lipoproteins: apoAI, apoB, and hsCRP Serum IL-6, TNF-α and LpPLA2G7	Lipids were measured on a Siemens analyzer. Lipoproteins were determined by immunonephelometric, using the Health Care Diagnostic Product on a Dade Behring nephelometer BNII System. Serum and interleukins, ELISA kits were used.	Clinical and radiographic examination	Asymptomatic apical periodontitis	In patients under 50, there was no significant increase in IL-6 and TNF-α compared to controls, but hsCRP and LpPLA2 levels were significantly higher. However, in patients over 50, there was a significant increase in IL-6, TNF-α, hsCRP, and LpPLA2 concentrations with aging.
Inchingolo et al.,**/**Italy**/**2013**/**Cohort	Case: 33 Control: 103	30-68 years	Total oxidant capacity Biological antioxidant potential (BAP)	Using a d-ROMs test kit Using a BAP test kit	Clinical and radiographic examination	Asymptomatic apical periodontitis	The patients with chronic apical periodontitis exhibited significantly higher levels of oxidative stress than control patients, as determined by the d-ROMs and BAP tests.
Cotti et al./Italy/2011/Cross-Sectional	Case: 20 Control: 20	20-40 years	IL-1, IL-2, IL-6, and TNF- α	Determined by ELISA test	Clinical and radiographic examination	Asymptomatic apical periodontitis	Patients with apical periodontitis with significantly greater blood concentrations of IL-1 (P <.05), IL-2 (P <.01), IL-6 (P <.05)
Abdolsamadi et al./Iran**/**2008/Case-Control	Case: 40 Control: 40	NR	IL-6	Determined by ELISA test	Clinical and radiographic examination	Asymptomatic apical periodontitis	Serum IL-6 concentration was significantly higher in the test group compared to the controls (P < 0.05).
Minczykowski et al./Poland/2001/Case-Control	Case: 20 Control: 20	Mean age: 33.8 years	Superoxide anion Hydrogen peroxide production	Gauging system in the non-stimulation state, with incubation, centrifugation, and absorption tested on free cells. Gauging system in the non-stimulation state, with incubation and centrifugation	Clinical and radiographic examination	NR	Non-stimulated cells from patients with chronic apical periodontitis exhibited significantly higher superoxide anion production and released greater amounts of H_2_O_2_ compared to control cells (P<0.001 and P<0.01 respectively).
Torabinejad/United States**/**1983**/**Case-Control	Case: 30 Control: 30	Case: mean age of 34.96 Control: mean age of 38.6	IgG, IgM, C3	Were determined using of commercially available radio-immunodiffusion (RID) plates.	Diagnosis made by radiographic images	Asymptomatic apical periodontitis	There was no statistical difference between the mean values of patients with periapical lesions and those of the control.
Sample: Gingival Crevicular Fluid							
Author/Country/Year/Study Design	Participants		Biomarker Evaluation		Apical Periodontitis		Results
	Sample size	Age mean (SD) in years	Biomarker	Evaluation Method	Diagnostic Method	Clinical Diagnosis	
Chile/cross-sectional/2019	Case: 49 Control: 13	NR	MMP -2 and -9; MMP -8; MPO; IL-1, IL-6, TNFa, Dkk-1, ON, PTN, TRAP-5 and OPG.	Gelatin zymography; ELISA, IFMA; ELISA; Multiplex detection panels.	Was defined by the presence of a radiographic apical lesion and negative clinical tests of pulp sensitivity.	Asymptomatic apical periodontitis	The MMP-9 and MMP-8 were higher in asymptomatic apical periodontitis (AAP), versus healthy individuals (p < 0.05). The highest diagnostic accuracies were observed for the active form of MMP-9 and MMP-8 (AUC > 0.90) in AAP.
Analysis Material: Saliva							
Author/Country/Year/Study Design	Participants		Biomarker Evaluation		Apical Periodontitis		Results
	Sample size	Age mean (SD) in years	Biomarker	Evaluation Method	Diagnostic Method	Clinical Diagnosis	
Haug & Marthinussen,**/**Norway**/**2019**/**Cross-sectional	Case: 42 Control: 39	> 20 years	Cortisol, CRP, IL-1ß, and IL-6.	Were analyzed using Salimetrics high-sensitivity salivary enzyme immunoassay (EIA) kits	Oral clinical examinations	Symptomatic apical periodontitis	Higher levels of cortisol, IL-1ß, and IL-6 and increased salivary flow were detected in patients with pain when compared to controls (P <.05). C-reactive protein (CRP) was higher in patients with acute pain compared to control participants without pain, but this difference was not statistically significant.
Sample: Blood/Serum							
Author/Country/Year/Study Design	Participants		Biomarker Evaluation		Apical Periodontitis		Results
	Sample size	Age mean (SD) in years	Biomarker	Evaluation Method	Diagnostic Method	Clinical Diagnosis	
Montis et al.,**/**Italy**/**2019**/**Case-control	Case: 11 Control: 8	Case: Mean age of 47 years Control: Mean age of 43.2 years	Salivary metabolites	Analyzed using an Agilent 5977B interfaced to the GC 7890B	Clinical and radiographic examination	Chronic apical abscess	The CAA group had significantly higher concentrations of 4-hydroxyhydrocinnamic acid, N-acetylneuraminic acid, inositol-like compounds, ornithine, putrescine, hypoxanthine, 5-aminopentanoic acid, proline, uracil, lysine, stearic acid, threonine, uric acid, glycine, and phosphoethanolamine compared to the control group. Conversely, the CAA group exhibited significantly lower concentrations of sorbitol, maltose, glucose, xylitol, succinic acid, ethanolamine, lactic acid, palmitic acid, citric acid, urea, 1,2-propanediol, and meso-2,3-butanediol than the control group.
Pietiäinen**/**Finland**/**2019**/**Cohorte	Case: 262 Control: 162	Mean age of 62.9 years	IgA and IgG	Determined by ELISA test	Clinical and radiographic examination	NR	Among serum or saliva IgA-class antibody levels, only sporadic significant differences were observed between patients with and without endodontic findings, whereas among IgG-class antibodies several significant differences were found.
Vengerfeldt et al.,**/**Estonia**/**2017**/**Case-Control	Case: 69 Control: 17	Mean age of 38.7 years	Total peroxide concentrations (TPX); Myeloperoxidase (MPO); 8-isoprostanes (8-EPI)	An OxyStat colorimetric assay kit was used for measuring TPX; ELISA test was used to determine MPO and measurement of 8-EPI.	Diagnosis was made by radiographic images	NR	The highest MPO and 8-EPI levels were seen in the case of pCAP and pulpitis, in saliva, while in sCAP and abscess patients these markers tended to display lower values. The highest levels of OSI were seen in pCAP and abscess patients, in saliva also in sCAP patients. The control group showed the lowest levels of all markers.
Sample: Blood/Serum							
Author/Country/Year/Study Design	Participants		Biomarker Evaluation		Apical Periodontitis		Results
	Sample size	Age mean (SD) in years	Biomarker	Evaluation Method	Diagnostic Method	Clinical Diagnosis	
Acronyms and abbreviations							
**Biomarkers**					**Analytical tests**		
**IL –** Interleukins **PAPP-A –** Pregnancy-associated protein-A **HsCRP –** High-sensitivity C-reactive protein **Ig –** Immunoglobulins **MMP –** Matrix metalloproteinases **sVCAM –** Soluble vascular cellular adhesion molecule **sE-selectin –** Soluble E-selectin **AOOP –** Oxidation protein products **PON1 –** Paraoxonase 1 **TRAP –** Total radical trapping antioxidant parameter **NO –** Nitric oxide **NOx –** Nitric oxide metabolites **LOOH –** Hydroperoxides **-SH –** Sulfhydryl **LpPLA –** Lipoproteins **BAP –** Biological antioxidant potential **TNF-α –** Tumor necrosis factor alpha **IC –** Immune complexes **C3 –** Complement component 3 **Dkk-1 –** Dickkopf-related protein **ON –** Osteonectin **PTN –** Periostin **TRAP –** Trate-resistant acid phosphatase **OPG –** Osteoprotegerin **CRP –** C-reactive protein **TPX –** Total peroxide concentrations **MPO –** Myeloperoxidase **EPI –** Isoprostanes					**ELISA –** Enzyme Linked Immuno Sorbent Assay **IFMA –** Time-resolved immunofluorometric method **EIA –** Enzyme immunoassay **Immuno kit –** Immunoassay kit		
					**Others**		
					**NR –** Not reported		

Furthermore, in all of the 17 evaluated studies, apical periodontitis was diagnosed by a combination of clinical and radiographic tests (6, 29–44.).

**Figure 5:**
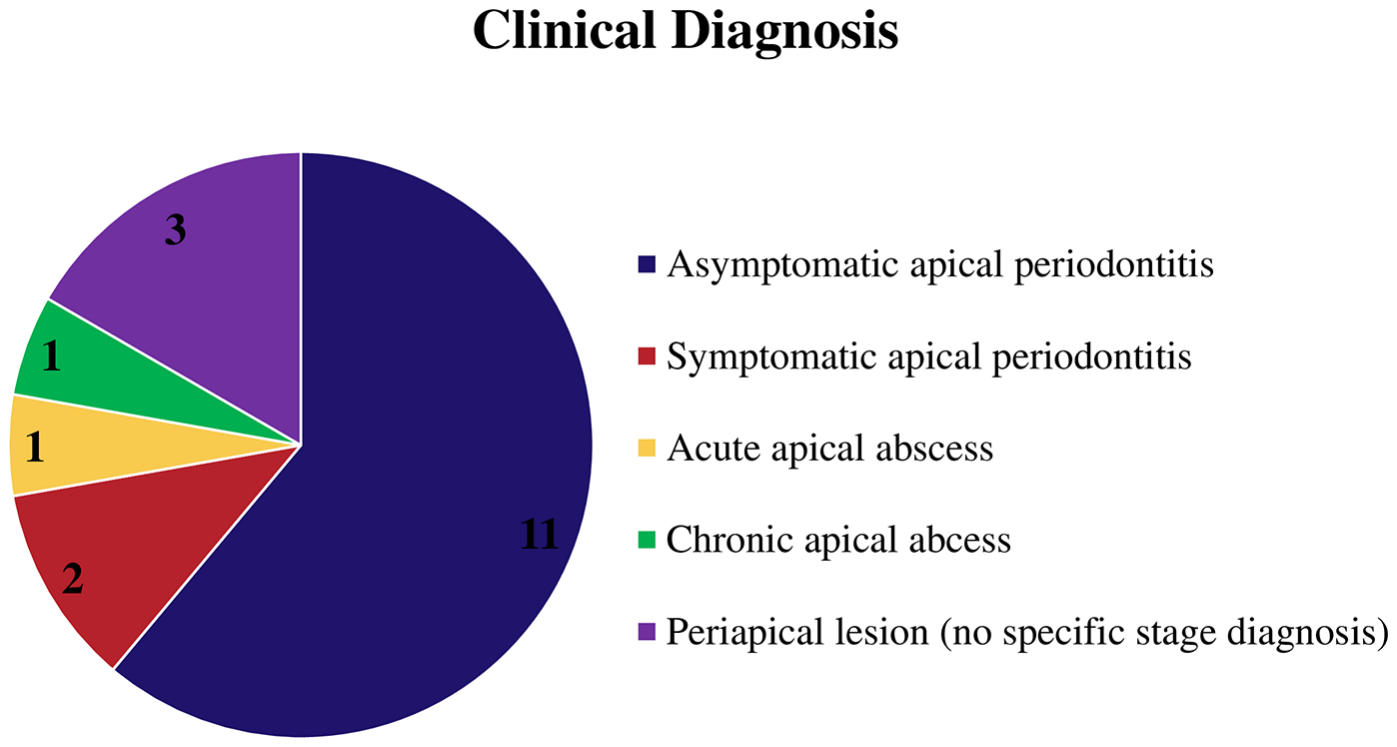
Graphic showing the clinical diagnosis of the studies.

Regarding clinical diagnosis, 11 studies specifically diagnosed asymptomatic AP (29–31, 33–35, 37, 39, 41, 43, 44). Some studies have specified the diagnosis of symptomatic apical periodontitis (36, 38), acute apical abscess (44), and chronic apical abscess (42). Additionally, three studies did not specify a diagnosis other than periapical lesions (6, 32, 40), as shown in **Figure 5**.

In terms of lesion extent, 12 articles were not specific (6, 29, 31, 32, 34–36, 38, 39, 41–43), three observed lesions >3 mm (30, 33, 40), and two observed lesions <2 cm (37, 44).

**Figure 6:**
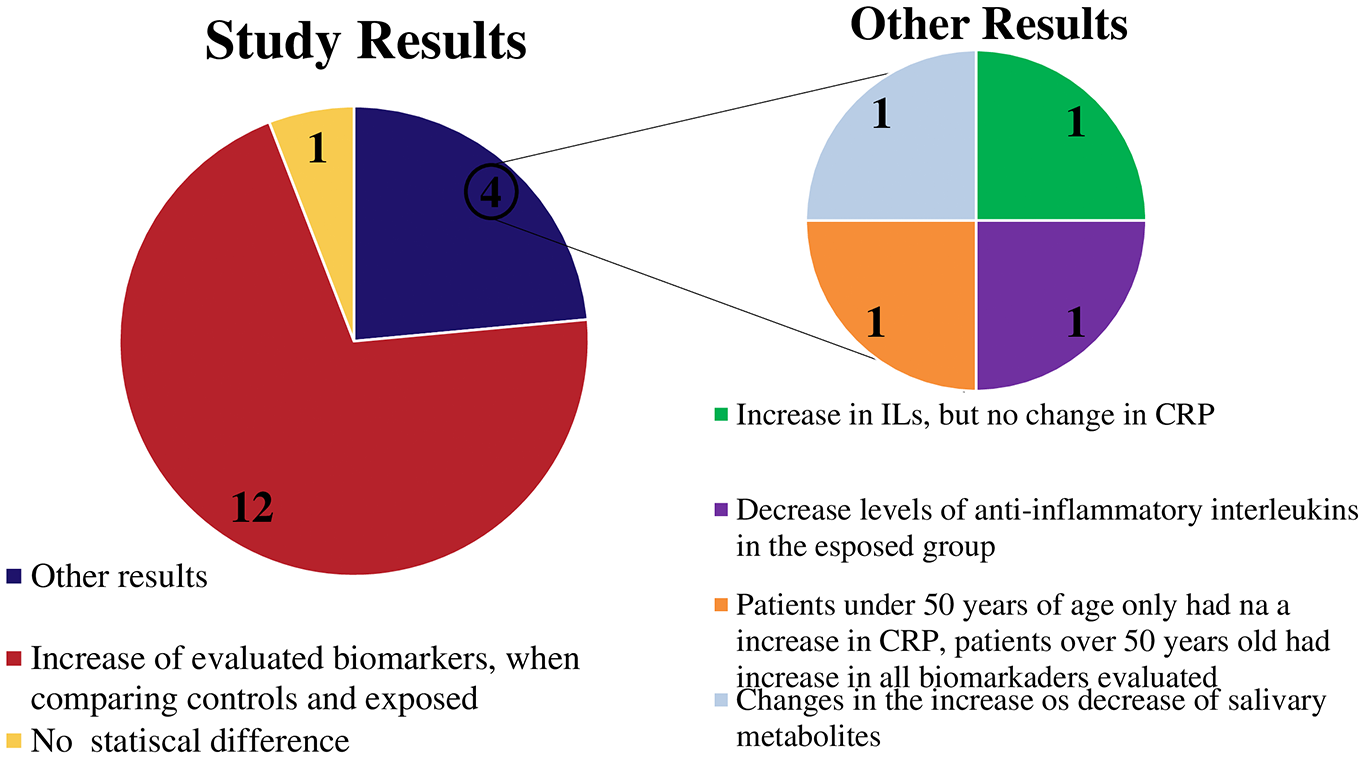
Graphic showing the main results of the studies.

Biomarker concentrations in individuals with apical periodontitis versus healthy patients were analyzed in 12 studies (6, 29–33, 35, 37–40, 44), revealing significant differences. However, the remaining four studies reported different outcomes. Rethnam Haug et al. (36) found a substantial increase in interleukins in the exposed group, but there was no significant difference in CRP concentration. Kimak et al. (41) showed that patients under 50 did not have a significant increase in TNF-α but a significant increase in LpPLA2 and HsCRP, while patients over 50 years had significant changes in all biomarkers tested. In addition, Georgiou (34) found a difference in the concentration of biomarkers with reduced levels of anti-inflammatory interleukins in the group with AP. Finally, Montis et al. (42) observed a significant difference in the concentrations of 76 salivary metabolites, some of which were identified at higher concentrations and others at lower concentrations, compared with the control group. Furthermore, Torabinejad et al. (43) found no significant differences in group comparisons (**Figure 6**).

### 3.3 Analyses of risk of bias

#### 3.3.1 Quality assessment and risk of bias

**Table 3** summarizes the findings of the methodological quality and bias risk assessments. As a result, ten studies were rated as high quality, with a low risk of bias (6, 29, 32, 34, 35, 38, 41–44); 1 as fair quality and medium risk of bias (43); and six as low quality with a high risk of bias (31, 33, 36, 37, 39, 40). The main issues found in the studies with a high risk of bias were the verification of exposure and non-response rates. Flaws in the adequate definition of cases, sample size, and ascertainment of exposure were observed in studies with a moderate risk of bias.

**Table 3:** Quality assessment and risk of bias.

CASE CONTROL	(29)	(32)	(35)	(34)	(41)	(40)
Selection						
1) Is the Case Definition Adequate?	*	*	*	*	*	*
2) Representativeness of the Cases	–	–	*	*	–	–
3) Selection of Controls	*	*	*	*	–	–
4) Definition of Controls	*	*	*	*	*	*
Comparability						
1) Comparability of Cases and Controls on the Basis of the Design or Analysis	*	*	*	**	*	*
Exposure						
1) Ascertainment of Exposure	*	*	*	*	*	*
2) Same method of ascertainment for cases and controls	*	*	*	*	*	
3) Non-Response Rate	–	–	–	*	–	–
CASE CONTROL	(42)	(38)	(43)		(44)	
Selection						
1) Is the Case Definition Adequate?	*	*			*	
2) Representativeness of the Cases		*			*	
3) Selection of Controls	*	*			*	
4) Definition of Controls		*	*		*	
Comparability						
1) Comparability of Cases and Controls on the Basis of the Design or Analysis	*	*	*		*	
Exposure						
1) Ascertainment of Exposure	*	*	*		*	
2) Same method of ascertainment for cases and controls	*	*	*		*	
3) Non-Response Rate						
CROSS-SECTIONAL	(30)	(31)	(33)		(36)	(39)
Selection (5 stars max)						
1) Is the Case Definition Adequate?	*					*
2) Sample			*			
3) Non-respondents	*	*	*		*	*
4) Ascertainment of the exposure (risk factor)	**	**	**		**	**
Comparability (2 stars max)						
1) The subjects in different outcome groups are comparable, based on the study design or analysis. Confounding factors are controlled	*	**	**		**	*
Outcome (3 stars max)						
1) Ascertainment of outcome	*	*	*		*	
2) Statistical test	*		*		*	*
COHORT STUDIES	(37)	(6)				
Selection						
1) Representativeness of the Exposed Cohort	*	*				
2) Selection of the Non-Exposed Cohort		*				
3) Ascertainment of Exposure						
4) Demonstration That Outcome of Interest Was Not Present at Start of Study	*	*				
Comparability						
1) Comparability of Cohorts on the Basis of the Design or Analysis	*	*				
Outcome						
1) Assessment of Outcome	*	*				
2) Was Follow-Up Long Enough for Outcomes to Occur	*	*				
3) Adequacy of FollowUp of Cohorts						

#### 3.3.2 Evaluation of control statements for possible confounders and bias consideration

Eight eligible studies (29, 32, 34, 37, 39, 40, 43, 44) had not performed adjusment analysis and four studies (31, 38, 41, 42) were unclear about the conducting or reporting of their confounding control analyses. Therefore, only five eligible studies (6, 30, 33, 35, 36) were selected to be critically appraised in further steps.

Two studies (6, 33) made a specific mention of the term “confounding,” and one study (35) alluded to it. Only one study (33) used the term “bias.” Two studies (30, 36) mentioned non-adjusted variables: gingival crevicular fluid in chronic periodontitis and asymptomatic apical periodontitis (30) and severity of stress among groups (36). Two other studies (6, 35) mentioned non-adjusted variables, such as uric acid and xanthine oxidase (35), intracanal bacterial samples, and serum cross-reactive antibodies (6). All five studies (6, 30, 33, 35, 36) mentioned that their results may be affected by confounders and stated the need for caution when interpreting their results. Only one study (30) had limitations regarding confounders in their conclusions. The results of the evaluation of the control statements for possible confounders and bias considerations are summarized in **Table 1**.

#### 3.3.3 Assessment of confounding factors

A total of 145 variables were identified in selected studies. There were 98 variables used in multivariate analysis to control possible confounders. No studies performed sample stratification. Only one study (36) used matching variables, which were “age” and “sex.”

Six confounding domains were identified in the selected studies: (1) biomarkers; (2) oral health-related domains; (3) body and comorbidities; (4) sociodemographic and socioeconomic status; (5) quality of life; and (6) analysis. The confounding domains identified in each study are listed in **Table 4**.

**Table 4:** Confounding domains identified in selected studies.

Confounding domains						
Author, year	*Biomarkers*	*Oral-health related*	*Body and comorbidities*	*Sociodemographic and socioeconomic*	*Quality of life*	*Measurements of analysis*
30	x	x	x	x	–	x
35	x	x	x	x	x	–
36	x	x	x	x	x	–
33	x	x	x	x	–	–
6	x	x	x	x	–	–

x, identified in the study; –, not identified in the study.

**Figure 7:**
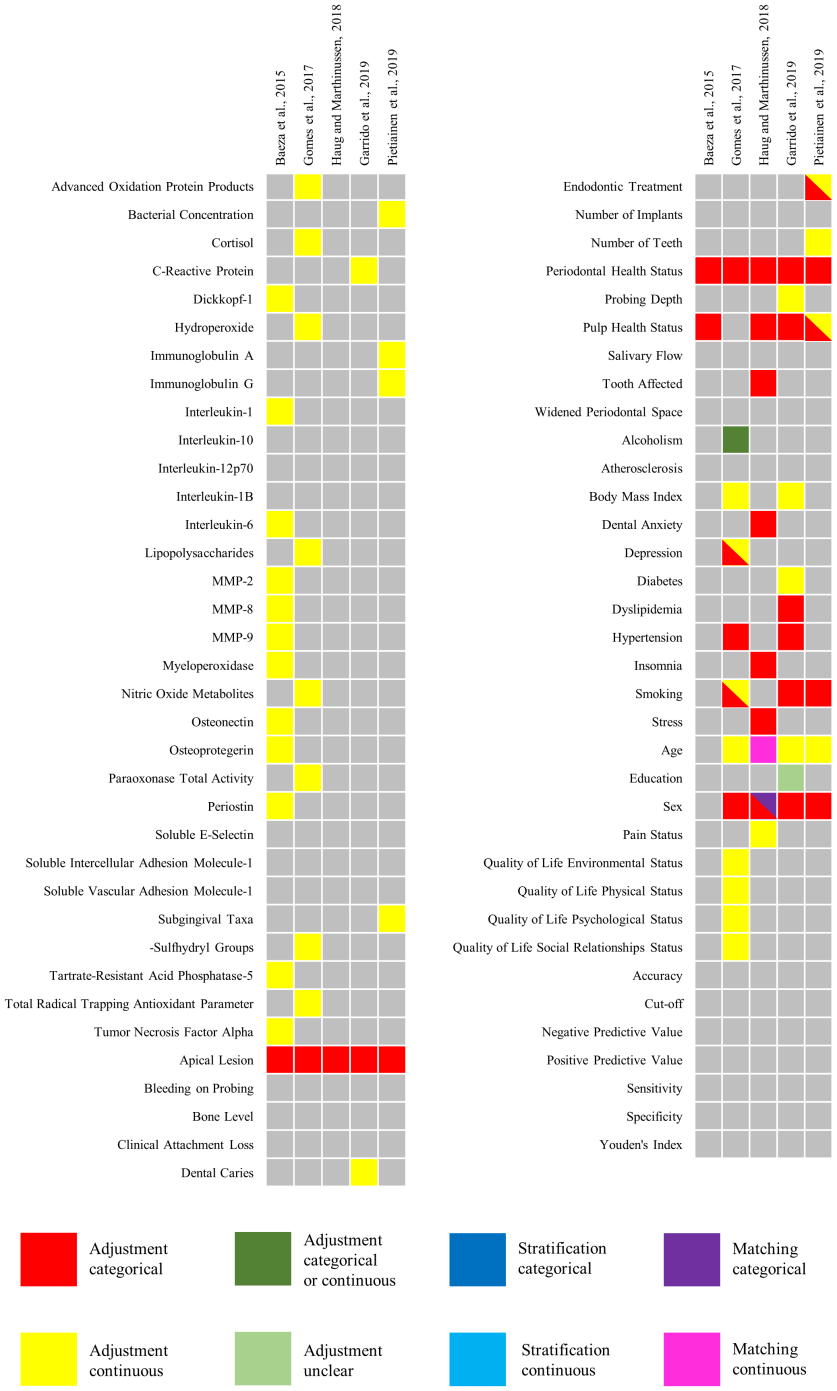
The results of the analysis of confounding factors in the eligible articles.

The “Biomarkers” domain was the most explored, with a total of 45 variables, whereas both the “quality of life” and “measurements of analysis” domains were the least explored, with seven variables in each of them. Descriptions and examples of the variables identified in each domain are provided in **Supplementary Table 3**. The results of the analysis of confounding factors in the five eligible articles (6, 30, 33, 35, 36) are shown in **Figure 7**.

### 3.4 Level of evidence (GRADE tool)

The GRADE narrative analysis was used to assess the quality of evidence regarding changes in biomarker levels in the blood/serum, gingival crevicular fluid, and saliva in apical periodontitis (**Table 5**). The observational studies included in the analysis provided evidence of moderate certainty regarding the three outcomes. However, some issues were identified in some of the studies. Three studies (41, 43) reported problems with sample selection in the assessment of changes in blood and serum biomarkers. Comparability issues across groups have been reported for changes in gingival crevicular fluid biomarkers. Similarly, all four studies (6, 36, 42, 44) that assessed the changes in salivary biomarkers had comparability issues across groups.

**Table 5:** The certainty of evidence: association between biomarkers and apical periodontitis.

Apical periodontitis compared to control for biomarkers Bibliography:							
Certainty assessment							Summary of findings
Participants (studies) Follow-up	Risk of bias	Inconsistency	Indirectness	Imprecision	Publication bias	Overall certainty of evidence	Impact
Changes in blood/serum biomarkers							
**925** **(12 observational studies)**	very serious^a^	not serious	not serious	not serious	all plausible residual confounding would reduce the demonstrated effect	⊕⊕⊕◯ Moderate	Adults presenting apical periodontitis, when compared to a control group, showed higher levels of High-sensitivity C-reactive protein (hsCRP), and multiple interleukins (IL-1, IL*-1β*, IL-2, IL-6), tumor necrosis factor-alpha (TNF-α), and immunoglobulins (IgG and IgM). In addition, these patients also showed a higher neutrophil/lymphocyte ratio (NLR) level, higher root canal lipopolysaccharide (LPS) levels, significantly higher levels of oxidative stress markers (e.g., superoxide anion), and higher levels of immune complexes among patients with chronic periapical granuloma and periapical cysts.
Changes in gingival crevicular fluid biomarkers							
**62** **(1 observational study)**	very serious^b^	not serious	not serious	not serious	all plausible residual confounding would reduce the demonstrated effect	⊕⊕⊕◯ Moderate	MMP-9 and MMP-8 were higher in asymptomatic apical periodontitis when compared to a control group.
Changes in saliva biomarkers							
**611** **(4 observational studies)**	very serious^c^	not serious	not serious	not serious	all plausible residual confounding would reduce the demonstrated effect	⊕⊕⊕◯ Moderate	Adults with apical periodontitis, when compared to a control group, showed higher levels of immunoglobulins (IgG), cortisol, C-reactive protein (CRP), interleukins (L-1ß, and IL-6), and an increased salivary rate. Also, specific salivary metabolites showed higher (e.g., 4-hydroxyhydrocinnamic acid, threonine, uric acid, and glycine) and lower (e.g., sorbitol, maltose, glucose, xylitol) concentrations in apical periodontitis groups when compared to controls.

CI, confidence interval.

Explanations

^a^Problems in the sample selection were observed in three studies (37, 41, 43).

^b^Problems in comparability across groups were detected.

^c^Problems in comparability across groups were detected in all four studies (6, 36, 42, 44).

## 4 Discussion

This systematic review revealed significant differences in the presence of biomarkers between patients with apical periodontitis compared to healthy individuals. This association was observed in 12 papers (6, 29–33, 35, 37–40, 44) included in this review, showing that systemic alterations may be triggered or aggravated in the presence of endodontic infection (45).

To consider systemic factors, only studies that evaluated biomarkers in body fluids were included because of recent advancements in the development of new methods for diagnosing and monitoring diseases by analyzing biomarkers in body fluids (46). Body fluids have a broad range of secretomes and various cell types that contribute to the analysis systems, increasing the spectrum of fluid-based analysis systems (47). Out of the 17 studies, 12 evaluated biomarkers in blood samples (29, 31–35, 37, 38, 39 40, 41, 41, 43), which are generally considered the best fluid to evaluate systemic processes through biomarkers (48, 49). Additionally, four studies evaluated biomarkers in saliva samples (6, 36, 42, 44). Saliva has become an alternative fluid to blood since salivary components are derived from both glands and blood owing to the high vascularity of salivary glands. Therefore, biomarkers in the saliva may reflect those in the blood (48). In addition, Baeza et al. (30) analyzed gingival crevicular fluid samples, which in a healthy state are considered a serum transudate as fluid from the surrounding capillaries that passes into the gingival sulcus and has a protein concentration similar to that of the interstitial fluid. However, in an inflamed state, it is considered an exudate with a protein concentration resembling to that of the serum (50).

All included studies had only clinical diagnoses available because they performed only radiographic examinations and clinical tests; however, a definitive diagnostic differentiation of the actual periapical status can only be attained by histopathology (51). In this review, 17 studies (6, 29–44.) diagnosed apical periodontitis by combining clinical and radiographic tests. This highlights the lack of adequate control for confounders in studies seeking an association between apical periodontitis and the systemic inflammatory burden.

Regarding the clinical diagnosis, most of studies included in this review specifically diagnosed asymptomatic apical periodontitis, which is a common type of periapical disease (29–31, 33–35, 37, 39, 41, 43, 44). However, other studies have focused on other types of periapical diseases such as symptomatic apical periodontitis (36, 38), acute apical abscess (44), and chronic apical abscess (42). In contrast, three studies did not specify a diagnosis other than “periapical lesion.”

Identifying the type of periapical disease is important, as it can affect the presentation of symptoms and the level of systemic involvement. For example, symptomatic infections occur when bacteria invade the periradicular tissues, leading to more noticeable symptoms (52). In contrast, asymptomatic apical periodontitis may not present with any symptoms. Additionally, different types of periapical diseases may lead to different levels of systemic involvement as endodontic infections can contribute to the overall oral infectious burden or lead to bacteremia stemming from endodontic treatment or acute abscesses. In addition, immunoglobulin levels can differ among different types of periapical lesions, with cysts exhibiting higher immunoglobulin levels than granulomas (53). Therefore, the specific diagnosis of periapical disease can play an important role in determining appropriate biomarkers to evaluate and interpret the results.

Thus, examining the biomarkers present in a systemic manner can help in the differential diagnosis of a specific type of periapical disease, as the cells and cytokines present tend to have a different predominance according to the type of lesion (5). Thus, the literature shows that the presence of T helper 2 (Th2) cells, Interleukin-1 (IL-1) and TNF-α, is more pronounced in periapical cysts, while T helper 1 (Th1) cells, Interleukin-10 (IL-10) and FoxP3 are more associated with the diagnosis of periapical granulomas (54, 55). In addition, IL-6, due to its pro-inflammatory character, is present in the earliest stages of development of symptomatic apical periodontitis (56). Furthermore, Interleukin-17 (IL-17) acts in the stimulation of Interleukin-8 (IL-8) production, which in turn aggravates the inflammatory process of apical periodontitis, and in the production of RANKL - which acts in the process of osteoclastogenesis, aggravating bone resorption; it is found mainly in cases of chronic apical periodontitis (57–59).

Biomarkers play a crucial role in providing valuable information regarding the status of periapical lesions, severity, degree of inflammation, and immune response within a clinical context. These biomarkers can be assessed in specimens such as blood, saliva, or tissue, offering insights into specific biological processes and distinguishing between the active and chronic stages of periapical lesions (60). Biomarkers linked to acute inflammation, such as proinflammatory interleukins (IL-1, IL-6, and IL-8) and C-reactive protein (CRP), are indicative of recent periapical lesions. Elevated levels of these biomarkers may suggest heightened and severe inflammation, providing valuable information about the severity of the lesion and the degree of the acute inflammatory response (61). Conversely, biomarkers associated with chronic inflammation can offer insight into persistent and long-standing periapical lesions. Elevated concentrations of pro-inflammatory cytokines, including interleukins and TNF-α, may signify intensified and active inflammation in these chronic lesions (62). The presence of heightened levels of immunoglobulins, such as IgG and IgA, indicates an adaptive immune response against chronic infection, potentially playing a role in immune defense mechanisms aimed at combating persistent inflammation (63). To substantiate these observations, comprehensive research involving the analysis of biomarker profiles in patients with varying stages of periapical lesions combined with clinical data and imaging assessments is warranted.

In this context, although the lesion size is not commonly considered in studies that evaluate correlations between endodontic disease and the presence of systemic damage, it is essential to assess this factor. Among the selected studies, 12 articles were not specific (6, 29, 31, 32, 34–36, 38, 39, 41–43), three observed lesions >3 mm (30, 33, 40), and two observed lesions <2 cm (37, 44). Matsuo et al. (64) evaluated the exudates collected from lesions and observed that the levels of IgG and IgA were directly proportional to the periapical lesion size. Although the selected studies did not directly evaluate the material collected from the lesion, this may indicate that lesion size can systemically interfere with biomarkers.

The presence of pro-inflammatory biomarkers, such as interleukins (e.g., IL-6 and IL-1β), in the bloodstream is positively correlated with the occurrence and intensity of systemic inflammation (39). The release of these pro-inflammatory cytokines indicates an activated immune response throughout the body in response to infections or chronic inflammatory conditions (5, 39). Elevated levels of systemic inflammatory markers like C-reactive protein (CRP) and tumor necrosis factor-alpha (TNF-α), are associated with the degree of systemic inflammation and may reflect the activity and severity of inflammation (5, 25). The presence of inflammatory biomarkers such as interleukins (e.g., IL-8), is positively correlated with the occurrence and intensity of local inflammation in saliva. These cytokines may indicate an inflammatory response at the site of origin, such as in oral infections or periodontal conditions (36, 65). Elevated levels of oxidative markers in the saliva, such as total peroxide concentration (TPX), myeloperoxidase (MPO), and 8-isoprostanes (8-EPI), suggest increased production of reactive oxygen species (ROS) and imply more intense and extensive inflammation associated with apical lesions (66). Conversely, the presence of immunoglobulins (IgA and IgG) in the saliva is associated with versatile adaptive immune responses against both local bacterial and host derived (6). Immunosuppressive biomarkers, like interleukin-10 (IL-10), show an inverse relationship with the severity of both systemic and local inflammations when detected in blood and saliva (36, 65). Higher IL-10 levels may signify an anti-inflammatory response and the modulation of the immune system (6).

In the presence of periapical infection, T cells, macrophages, and other cells produce chemicals that increase osteoclast production (RANKL expression). Proinflammatory cytokines (such as TNF-α, IL-6, IL-11, IL-17, and IL-1β) and chemical mediators (prostaglandins and bradykinins) are released. These substances accumulate at high concentrations in the bone and decrease osteoprotegerin expression in bone marrow stromal cells (59, 67). Among the selected studies, these and other biomarkers involved in periapical lesion pathogenesis were identified among the selected studies. Interleukins were found in nine studies (29–34, 36, 39, 41), followed by oxidative markers in five studies (30, 35, 37, 40, 44), immunoglobulins in four articles (6, 33, 38, 43), C-reactive protein in five articles each (33, 34, 36, 38, 41), and TNF-α in five studies (30–32, 34, 41) in addition to matrix metalloproteinase, lipoproteins, and lipids.

The limitations of the observational design of the eligible studies should be acknowledged. First, observational studies may be influenced by confounding factors, which can be mitigated by controlling known confounders using multivariate analyses (68). However, unknown confounders that are not controlled can significantly affect data interpretation, limiting the ability to make cause-and-effect statements from observational studies (10). Therefore, it is important to explicitly conduct and report adjustment analyses in the critical appraisal of eligible studies, and other steps serve as tools for analyzing the remaining studies (6, 30, 33, 35, 36).

While all the studies that remained in the analysis (6, 30, 33, 35, 36) mentioned the need for caution when interpreting their results, three studies (6, 33, 36) did not explicitly state this. This lack of explicit mention may result in a misinterpretation of the feasibility and generalizability of the study for the average reader. Although some studies (33, 35) have the aforementioned limitations, it is common for readers to focus solely on the final section. Therefore, it is important to include brief limitations and cautions in the study conclusions to provide a more complete understanding of the results.

There was a good proportion of controlled variables in the critically appraised studies (98/145), however, three identified domains were almost unexplored: “sociodemographic and socioeconomic,” “quality of life,” and “measurements of analysis.” Although these studies focused on sample analyses for biomarkers, these samples were collected from study participants and may have indirect or direct effects on the environmental factors of daily living. Additionally, laboratory tests have different aspects depending on the test used and the conditions under which they were performed. This supports the need to explicitly acknowledge more variables within the unexplored domains, which does not necessarily invalidate the results of each study. Notably, there can also be more confounding domains that are not yet identifiable, highlighting the need for caution when interpreting observational study results.

The quality of evidence for the association between biomarkers and apical periodontitis can be considered moderate using the GRADE approach, which implies that there are some limitations to the available evidence. This review included a substantial number of studies that showed significant differences in the presence of biomarkers between patients with and without apical periodontitis. However, there are some limitations, such as the reliance on clinical diagnosis rather than histopathology, which may have influenced the accuracy of the diagnosis. Moreover, these studies used different fluids to measure biomarkers, which may have affected the consistency of the results. Therefore, while describing the quality of evidence, it is important to provide a balanced assessment and highlight any limitations that could affect the interpretation of results.

Moreover, based on the findings of this systematic review, it is recommended for further research involves stratifying patients into those with apical periodontitis and those without. By comparing biomarker levels in these two groups separately, researchers can elucidate whether the observed associations are specific to apical periodontitis or if they are influenced by the presence of others concurrent oral disease, such as periodontal disease. This approach would improve the precision and validity of future investigations into the systemic impact of apical periodontitis. Additionally, to address the observed heterogeneity in the selected studies regarding biomarker types and sample sources (blood plasma, saliva, gingival crevicular fluid), future studies should adopt standardized methodologies to enhance result comparability and generalizability.

Furthermore, a recommendation to conduct studies comparing the levels of biomarkers before and after endodontic treatment can provide valuable insights into the systemic effects of this dental procedure. By assessing biomarker levels before the initiation of endodontic treatment and comparing them with post-treatment levels, researchers can evaluate the impact of the treatment on systemic inflammation and overall health. This comparison can help determine whether endodontic treatment leads to changes in systemic biomarker levels, indicating potential systemic benefits or risks associated with the procedure. Additionally, such studies can contribute to a better understanding of the systemic implications of endodontic treatment and guide clinical decision-making to optimize patient outcomes and overall health.

## 5 Conclusion

The reviewed studies demonstrate a correlation between the presence of biomarkers in systemic fluids and apical periodontitis. Biomarkers associated with inflammation, such as IL-1, IL-2, and IL-6, as well as oxidative markers, including nitric oxide, superoxide anions, and immunoglobulins IgG and IgM, have been identified during the disease. Nonetheless, there was considerable diversity in the methodologies and materials employed for analysis between the studies. Therefore, further clinical investigations with standardized evaluation parameters are necessary to provide more accurate knowledge of the association between biomarkers and apical periodontitis.

Nevertheless, although our review finds a non-causal association between AP and elevated systemic biomarker levels, it is crucial to acknowledge the interrelationship between oral health and general health. Apical periodontitis, as part of the oral inflammatory burden, may impact systemic health, particularly when concurrent with other oral inflammatory conditions such as periodontal disease. Therefore, we underscore the importance of implementing effective preventive and therapeutic strategies for periodontal diseases and AP, aiming not only at oral health but also at mitigating the risk of systemic complications arising from oral inflammation.

## Data availability statement

The original contributions presented in the study are included in the article/**Supplementary Material**. Further inquiries can be directed to the corresponding author.

## Author contributions

JM-S: Conceptualization, Investigation, Methodology, Software, Writing – original draft. VC: Conceptualization, Investigation, Methodology, Software, Writing – original draft. DF: Conceptualization, Formal analysis, Investigation, Software, Supervision, Writing – review & editing. LB: Conceptualization, Formal analysis, Supervision, Validation, Visualization, Writing – review & editing. JM: Conceptualization, Formal analysis, Investigation, Supervision, Validation, Visualization, Writing – review & editing. CM: Investigation, Methodology, Software, Writing – review & editing. GM-V: Investigation, Methodology, Software, Supervision, Validation, Visualization, Writing – review & editing. NF: Conceptualization, Formal analysis, Investigation, Methodology, Software, Supervision, Validation, Visualization, Writing – review & editing. LP: Formal analysis, Methodology, Software, Supervision, Validation, Visualization, Writing – review & editing. LM: Conceptualization, Data curation, Formal analysis, Investigation, Software, Supervision, Validation, Visualization, Writing – review & editing. MM: Data curation, Formal analysis, Methodology, Supervision, Validation, Visualization, Writing – review & editing. RL: Conceptualization, Data curation, Formal analysis, Funding acquisition, Investigation, Methodology, Project administration, Resources, Supervision, Validation, Visualization, Writing – review & editing.
