# Supplementary material for: Is there any association between the presence of biomarkers and apical periodontitis? A systematic review
Source: Front Immunol. 2024 May 22;15:1366954. doi: 10.3389/fimmu.2024.1366954 (PMC11150667; doi:10.3389/fimmu.2024.1366954)
Supplement: Supplementary file 1 [file DataSheet_1.docx]

Supplementary Material

# Supplementary Tables

**Supplementary table 1:** Table with the MESHs and entryterms used in the search key for each database in title, abstract or keywords.

| SEARCH STRATEGY | | | |
| --- | --- | --- | --- |
|  | **MESHS** | **KEYWORDS/ENTRYTERMS** | |
| P | **Humans** | - Homo sapiens - Man (Taxonomy) - Man, Modern | - Modern Man - Human |
|  | Adult | Adults |  |
| E | Periapical Periodontitis | Periapical Periodontitides  Periodontitides, Periapical  Periodontitis, Periapical  Periodontitis, Apical  Apical Periodontitides  Apical Periodontitis  Periodontitides, Apical  Periodontitis, Acute Nonsuppurative  Acute Nonsuppurative Periodontitides | Acute Nonsuppurative Periodontitis  Nonsuppurative Periodontitides, Acute  Nonsuppurative Periodontitis, Acute  Periodontitides, Acute Nonsuppurative |
|  | Periapical Granuloma | Granuloma, Periapical  Granulomas, Periapical  Periapical Granulomas  Periapical Periodontitis, Chronic Nonsuppurative  Periodontitis, Apical, Chronic Nonsuppurative | Dental Granulomas  Granulomas, Dental  Dental Granuloma  Granuloma, Dental |
|  | Radicular Cyst | Cyst, Radicular  Cysts, Radicular  Radicular Cysts  Periapical Cyst  Cyst, Periapical  Cysts, Periapical  Periapical Cysts | Periodontal Cyst, Apical  Apical Periodontal Cyst  Apical Periodontal Cysts  Cyst, Apical Periodontal  Cysts, Apical Periodontal  Periodontal Cysts, Apical |
|  | Periapical Diseases | Disease, Periapical  Diseases, Periapical  Periapical Disease |  |
|  | Periapical Abscess | (“Dentoalveolar Abscess, Apical”)  Abscess, Apical Dentoalveolar  Abscesses, Apical Dentoalveolar  Apical Dentoalveolar Abscess  Apical Dentoalveolar Abscesses  Dentoalveolar Abscesses, Apical  Periodontitis, Apical, Suppurative  Periapical Periodontitis, Suppurative  Periapical Periodontitides, Suppurative  Periodontitides, Suppurative Periapical  Periodontitis, Suppurative Periapical  Suppurative Periapical Periodontitides  Suppurative Periapical Periodontitis  Alveolar Abscess, Apical Abscess, Apical Alveolar | Abscesses, Apical Alveolar  Alveolar Abscesses, Apical  Apical Alveolar Abscess  Apical Alveolar Abscesses  Abscess, Periapical  Abscesses, Periapical  Periapical Abscesses  Acute alveolar abscess;  Chronic alveolar abscess;  Pericemental abscess;  Acute apical abscess;  Chronic apical abscess;  Chronic dentoalveolar abscess;  Acute dentoalveolar abscess;  Phœnix abscess;  Acute periradicular abscess;  Chronic periradicular abscess |
| O | Biomarkers | Markers, Biological  Biologic Markers  Markers, Biologic  Biologic Marker  Marker, Biologic  Marker, Biological  Biological Marker  Biological Markers  Markers, Laboratory  Laboratory Markers  Laboratory Marker  Marker, Laboratory  Serum Markers  Markers, Serum  Marker, Serum  Serum Marker  Surrogate End Point  End Point, Surrogate  Surrogate Endpoint  Endpoint, Surrogate  Markers, Clinical  Clinical Markers  Clinical Marker  Marker, Clinical | Surrogate Endpoints  Endpoints, Surrogate  Surrogate End Points  End Points, Surrogate  Viral Markers  Markers, Viral  Viral Marker  Marker, Viral  Biochemical Marker  Biochemical Markers  Markers, Biochemical  Marker, Biochemical  Markers, Immunologic  Immune Markers  Markers, Immune  Marker, Immunologic  Immunologic Markers  Immune Marker  Marker, Immune  Immunologic Marker  Surrogate Markers  Markers, Surrogate  Marker, Surrogate  Surrogate Marker |
|  | Inflammation Mediators | Mediators, Inflammation  Mediators of Inflammation |  |
|  | Immunity, Active | Active Immunities  Active Immunity  Immunities, Active  Active Immune Response  Active Immune Responses | Immune Response, Active  Immune Responses, Active  Response, Active Immune  Responses, Active Immune |
|  | Blood Biomarkers | Blood Biomarkers |  |

**Supplementary table 2:** Table for risk of bias results.

| **OVERALL** | |
| --- | --- |
| **GOOD QUALITY** | 3 or 4 stars in selection domain AND 1 or 2 stars in comparability domain AND 2 or 3 stars in outcome/exposure domain |
| **FAIR QUALITY** | 2 stars in selection domain AND 1 or 2 stars in comparability domain AND 2 or 3 stars in outcome/exposure domain |
| **POOR QUALITY** | 0 or 1 star in selection domain OR 0 stars in comparability domain OR 0 or 1 stars in outcome/exposure domain |

**Supplementary table 3:** Description of confounding domains identified in selected studies.

|  | **Confounding domain** | **Description** | **Examples identified in selected studies** |
| --- | --- | --- | --- |
| 1 | Biomarkers | Biological markers and metabolites analyzed in samples collected from study participants. | AOPP; Bacterial Concentration; Cortisol; C-Reactive Protein; Dickkopf-1; Hydroperoxide; Immunoglobulin A; Immunoglobulin G; Interleukin-1; Interleukin-10; Interleukin-12p70; Interleukin-1B; Interleukin-6; Lipopolysaccharides; MMP-2; MMP-8; MMP-9; Myeloperoxidase; NOx; Osteonectin; Osteoprotegerin; Paraoxonase Total Activity; Periostin; Soluble E-selectin; -Sulfhydryl Groups; sICAM-1; sVCAM-1; Subgingival Taxa; TNFa; TRAP-5; TRAP. |
| 2 | Oral-health related | Clinical, radiographic, and other parameters related to the oral health. | Apical Lesion; Apical Lesion Size; Bone Level; Bleeding on Probing; Clinical Attachment Loss; Carious Teeth; DMFT Index; Endodontic Lesion Score; Endodontic Treatment Score; Gingivitis; Periodontal Health; Periodontitis; Number of Implants; Number of Teeth; Probing Depth; Root Canal Fillings; Salivary Flow; Symptomatic Pulpitis; Tooth Affected; Widened Periodontal Space. |
| 3 | Body and comorbidities | Clinical parameters and conditions related to the systemic health. | ASSIST-Alcohol; ASSIST-Hypnotics; Body Mass Index; Waist Circumference; Dental Anxiety; Diabetes; HbA1c; Dyslipidemia; Cholesterol; Triglycerides; Hypertension; Diastolic Pressure; Systolic Pressure; Insomnia; Major depression; BDI; HDRS; PDAY Score; Self-Reported Stress; Smoking; Cigarette Month-Packs/Year; Fagerstrom Total Score. |
| 4 | Sociodemographics and socioeconomic | Sociodemographic and socioeconomic aspects. | Age; Education; Sex. |
| 5 | Quality of life | Questionnaires and scores related to impacts on quality of life. | Days with Pain; NRS-Pain; WHOQoL-BREF; WHO-Environmental; WHO-Physical; WHO-Psychological; WHO-Social Relationships. |
| 6 | Measurements of analysis | Measurements related to the test analyses performed with samples collected from study participants. | Accuracy; Cut-off; NPV; PPV; Sensitivity; Specificity; Youden’s Index. |
| Acronyms and abbreviations | | AOPP - Advanced Oxidation Protein Products; MMP-2 - Matrix Metalloproteinase-2; MMP-8 - Matrix Metalloproteinase-8; MMP-9 - Matrix Metalloproteinase-9; NOx - Nitric Oxide Metabolites; sICAM-1 - Soluble Intercellular Adhesion Molecule-1; sVCAM-1 - Soluble Vascular Adhesion Molecule-1; TNFa - Tumor Necrosis Factor Alpha; TRAP-5 - Tartrate-Resistant Acid Phosphatase-5; TRAP - Total Radical Trapping Antioxidant Parameter; DMFT - Decayed, Missing, and Filled Teeth; ASSIST - Alcohol, Smoking, and Substance Involvement Screening Test; HbA1c - Glycated Hemoglobin; BDI - Beck's Depression Inventory; HDRS - Hamilton Depression Rating Scale; PDAY - Pathobiological Determinants of Atherosclerosis in Youth; NRS - Numerical Rating Scale; WHOQoL-BREF - World Health Organization Quality of Life Brief Version; NPV - Negative Predictive Value; PPV - Positive Predictive Value. | |
